# Modulation of the Oxidation End‐Product Toward Polysulfides‐Free and Sustainable Lithium‐Pyrite Thermal Batteries

**DOI:** 10.1002/advs.202205888

**Published:** 2023-01-05

**Authors:** Yang Jin, Hongfei Lu, Nawei Lyu, Di Zhang, Xin Jiang, Bin Sun, Kai Liu, Hui Wu

**Affiliations:** ^1^ Research Center of Grid Energy Storage and Battery Application School of Electrical and Information Engineering Zhengzhou University Zhengzhou Henan 450001 P. R. China; ^2^ State Key Laboratory of Alternate Electrical Power System with Renewable Energy Sources School of New Energy North China Electric Power University Beijing 102206 P. R. China; ^3^ State Key Lab of New Ceramics and Fine Processing School of Materials Science and Engineering Tsinghua University Beijing 100084 P. R. China

**Keywords:** FeS_2_, high‐temperature batteries, lithium iron disulfide batteries, LLZTO, solid‐state batteries

## Abstract

The FeS_2_ has abundant reserves and a high specific capacity (894 mAh g^−1^), commonly used to fabricate Li‐FeS_2_ primary batteries, like LiM_x_‐FeS_2_ thermal batteries (working at ≈500 °C). However, Li–FeS_2_ batteries struggle to function as rechargeable batteries due to serious issues such as pulverization and polysulfide shuttling. Herein, highly reversible solid‐state Li‐FeS_2_ batteries operating at 300 °C are designed. Molten salt‐based FeS_2_ slurry cathodes address the notorious electrode pulverization problem by encapsulating pulverized particles in time with e^−^ and Li⁺ flow conductors. In addition, the solid electrolyte LLZTO tube serves as a hard separator and fast Li^+^ channel, effectively separating the molten electrodes to construct a liquid–solid–liquid structure instead of the solid–liquid–solid structure of LiM_x_‐FeS_2_ thermal batteries. Most importantly, these high‐temperature Li–FeS_2_ solid‐state batteries achieve FeS_2_ conversion to Li_2_S and Fe at discharge and further back to FeS_2_ at charge, unlike room‐temperature Li‐FeS_2_ batteries where FeS and S act as oxidation products. Therefore, these new‐type Li‐FeS_2_ batteries have a lower operating temperature than Li‐FeS_2_ thermal batteries and perform highly reversible electrochemical reactions, which can be cycled stably up to 2000 times with a high specific capacity of ≈750 mAh g^−1^ in the prototype batteries.

## Graphical abstract

This work reports Li–FeS_2_ secondary thermal batteries (240–300 °C) with a liquid–solid liquid structure. The liquid lithium anode and FeS_2_ slurry cathode are isolated using a U‐shaped LLZTO tube. The high battery reversibility is attributed to the reversible transition of Fe and Li_2_S to FeS_2_ at high temperatures and FeS_2_ pulverization. The FeS_2_ regeneration also avoids the production of polysulfides.

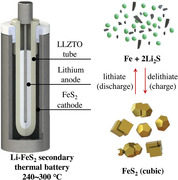

## 1 Introduction

The rapid development of large‐scale energy storage, electric vehicles, and aerospace continue to put forward higher requirements for the performance of lithium‐ion batteries.^[^ ^1^, ^2^, ^3^ ^]^ Unlike LiFePO_4_ (170 mAh g^−1^), LiCoO_2_ (140 mAh g^−1^), and other conventional insertion compounds, the iron disulfide (FeS_2_) is based on conversion reaction and has a higher theoretical specific capacity (894 mAh g^−1^).^[^ ^4^, ^5^, ^6^ ^]^ Besides, FeS_2_ material is nontoxic, harmless, environmentally friendly, and can be obtained directly from pyrite, which has an abundant reserve in nature. FeS_2_ can be paired with a lithium metal anode (3860 mAh g^−1^) to form a high‐energy‐density Li‐FeS_2_ battery whose energy densities can reach 995 Wh kg^−1^ and 1550 Wh L^−1^.^[^ ^6^ ^]^ In practice, the LiM_x_ alloy–FeS_2_ primary thermal battery has been widely used in military weapons due to its excellent high‐temperature electrochemical performance.^[^ ^7^ ^]^ However, the Li‐FeS_2_ batteries, whether working at room temperature or high temperature, are hard to work as rechargeable batteries as their poor reversibility or structural restriction, which dramatically limits the further application of the Li‐FeS_2_ batteries.

Tremendous efforts and improvements have been made to push Li‐FeS_2_ batteries to reversible in recent years, which are mainly FeS_2_ cathode materials modifications, including producing porous structures,^[^ ^8^, ^9^ ^]^ making better conductive carbon structures,^[^ ^10^, ^11^, ^12^, ^13^ ^]^ introducing excessive metal dopants,^[^ ^14^ ^]^ sulfur defects,^[^ ^15^ ^]^ and using sulfur doping.^[^ ^8^, ^16^ ^]^ Progress in electrolyte modification reduced the solubility of polysulfides and improved the interfacial compatibility with lithium metal anodes.^[^ ^17^, ^18^, ^19^, ^20^ ^]^ Although these methods have improved the reversibility and cycle performance of FeS_2_ materials to a certain extent, the high capability, long‐term cycles, and sufficient capacity retention are still challenging. The huge fluctuation in the shape and volume of the cathode material based on the conversion reaction will lead to serious issues, such as the pulverizing and shedding of the electrode and the dissolution and shuttling of polysulfides,^[^ ^6^, ^21^, ^22^ ^]^ which are ineradicable in the traditional organic electrolyte‐based system.

The reaction mechanism of the Li–FeS_2_ battery has been well studied. A consensus is that the final reduction products are Fe and Li_2_S.^[^ ^23^ ^]^ In addition, much research proved that the final oxidation products are FeS (hexagonal) and S under ambient conditions. That is, the battery's reaction in the first cycle would be different from that after. Nevertheless, the complex lithium‐iron disulfide reaction remains two major controversies: i) the presence of intermediate Li_2_FeS_2_; and ii) the reversibility of FeS_2_. The Li_2_FeS_2_ is a metastable product, and there is no direct observational evidence in the cathode detection of Li–FeS_2_ batteries. Even its experimental synthesis requires a high temperature of 900 °C and a long period of 16 h.^[^ ^24^ ^]^ At present, through some in situ battery observation experiments, it is believed that this hypothetical intermediate Li_2_FeS_2_ may not exist. the battery charge undergoes two‐step processes: Fe + Li_2_S → FeS; and then, Li_2_S→S.^[^ ^5^, ^25^ ^]^ The FeS is formed on the Li_2_S surface via heteroepitaxial growth.^[^ ^5^ ^]^

For controversy (ii), researchers have recognized that the FeS_2_ structure is not recoverable at 20–100 °C.^[^ ^26^, ^27^, ^28^ ^]^ Researchers were eager to achieve the reproduction of FeS_2_ after cycling at a high temperature (>400 °C).^[^ ^29^ ^]^ The researchers analyzed this was feasible from the results of calorimetric measurements of chemically delithiated Li_2_FeS_2_ and speculated the following reaction: FeS (hexagonal) + S$\overset{197^{\circ }C}{\to }$FeS_2_ (orthorhombic)$\overset{>265{\;}^{\circ }C}{\to }$FeS_2_ (cubic).^[^ ^27^ ^]^ However, in the past, high‐temperature Li‐FeS_2_ batteries could only rely on molten salt electrolytes. This kind of battery is essentially a primary battery and cannot recharge.^[^ ^30^ ^]^ So the regeneration of FeS_2_ during battery cycling has never been realized. Moreover, the high operating temperature (≈500 °C) of LiM_x_ alloy–FeS_2_ thermal batteries tends to cause thermal decomposition of FeS_2_, resulting in low capacity and discharge voltage. Applying pure lithium metal instead of lithium alloy as the anode can solve these problems. However, it is impossible to fix a liquid lithium anode (melting point: 180 °C) in a traditional FeS_2_ thermal battery with a solid–liquid–solid structure.^[^ ^31^ ^]^ Emerging solid‐state batteries are considered to be a useful framework for Li‐FeS_2_ batteries. FeS_2_ regeneration in high‐temperature solid‐state batteries may help the battery achieve high reversibility.

This paper reports a highly reversible solid electrolyte‐based Li‐FeS_2_ secondary thermal battery. The newly designed Li–FeS_2_ battery uses a liquid–solid–liquid structure, composing a FeS_2_ slurry cathode, a U‐shaped LLZTO solid electrolyte tube,^[^ ^32^, ^33^, ^34^, ^35^ ^]^ and a molten lithium metal anode, which is different from the solid–liquid–solid structure of previous Li–FeS_2_ batteries. This Li–FeS_2_ secondary thermal battery can solve the pulverizing issue of FeS_2_ particles by using the flowing Li^+^ and e^−^ conductive slurry in the cathode. The particle pulverized in this new battery further improved the effective contact area with the conductive network. Further research revealed that the electrochemical mechanism of the Li–FeS_2_ secondary thermal battery is radically different from that of a room‐temperature Li–FeS_2_ battery. The final oxidation product of the solid electrolyte‐based Li–FeS_2_ battery is FeS_2_ at 300 °C rather than FeS and S at room temperature. The reversible reaction from Li_2_S and Fe to FeS_2_ can avoid the formation of polysulfides.^[^ ^5^ ^]^ The assembled cells can effectively cycle 2000 times at a rate of 5 C (≈4.5 A g^−1^) and has a reversible specific capacity of ≈750 mAh g^−1^, together with a stable second discharge platform of ≈1.6 V. The proposed Li–FeS_2_ battery successfully pushed the Li–FeS_2_ battery from a primary battery to a secondary battery and realized a highly reversible cycle, which provides a new road for developing Li–FeS_2_ secondary batteries.

## 2 Results

### 2.1 The Structure and Basic Performance of Li–FeS_2_ Secondary Thermal Battery

**Figure 1:**
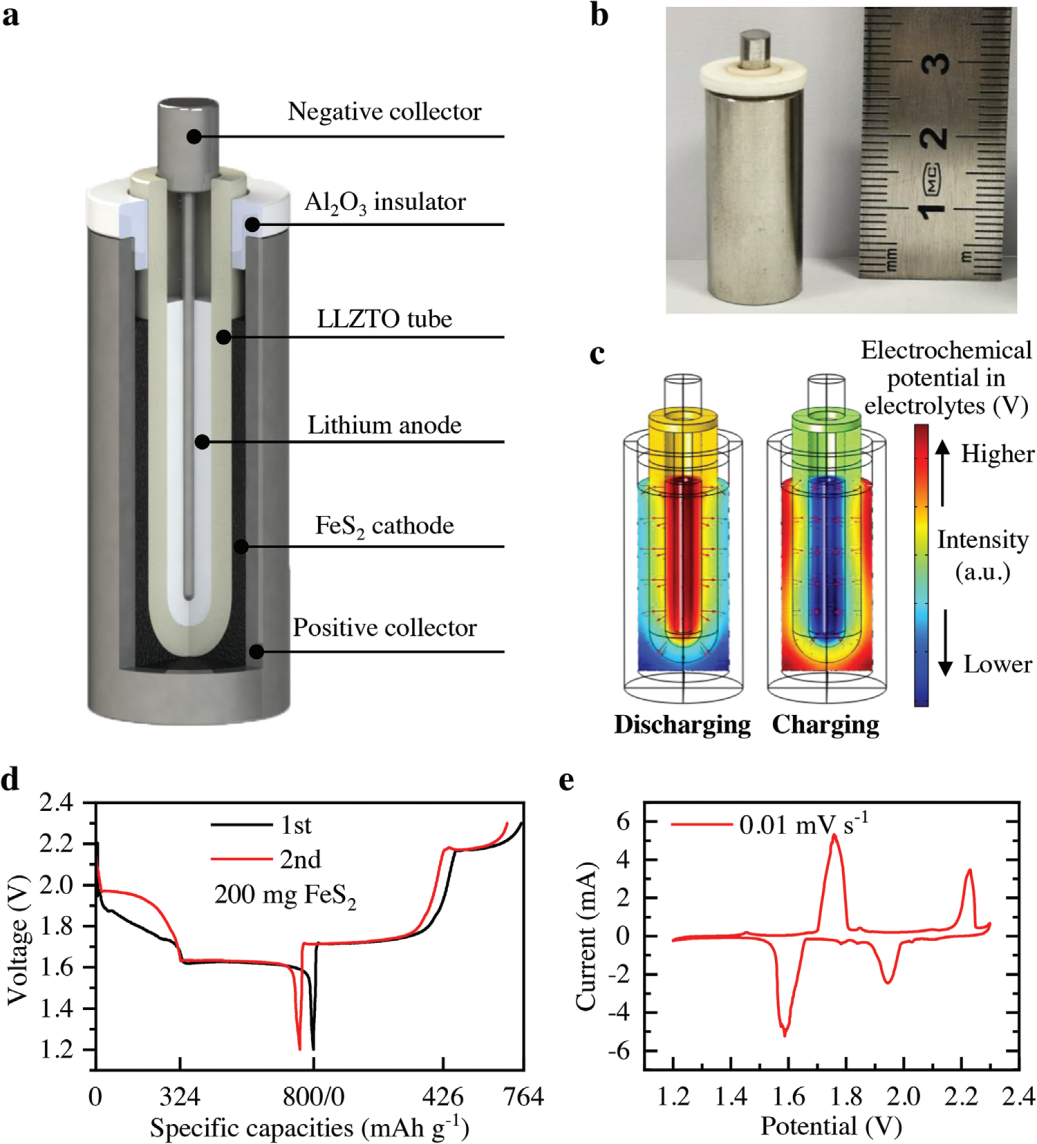
Solid electrolyte‐based Li–FeS_2_ secondary thermal battery. a) Schematic of the liquid–solid–liquid structure of the battery. b) An optical image of a small self‐made battery. c) Numerical simulation results of electrochemical potential in electrolytes and current density vector during battery discharging or charging. d) The first and second cycles of discharge–charge curves. e) Cyclic voltammetry curve at 0.01 mV s^−1^.

As shown in **Figure** **1**a,b, a solid electrolyte‐based Li‐FeS_2_ secondary thermal battery mainly comprises five parts: FeS_2_ cathode, U‐shaped LLZTO tube (Figure S1, Supporting Information), lithium metal anode, positive collector, and negative collector. The self‐made 316L battery case stores high‐temperature electrode materials and draws current as a positive collector. The Al_2_O_3_ insulator keeps the LLZTO tube in the center of the battery case. A stainless‐steel rod is inserted into the ceramic tube as a negative current collector. When the battery temperature exceeds 217 °C, the FeS_2_ cathode is a flowing slurry comprising FeS_2_, CNTs, and low melting point lithium salts (Figure S2, Supporting Information). Specifically, CNTs provide a good e^−^ conductive network, and lithium salts provide fast Li^+^ conductive pathways and a fluid environment. The lithium salts adopt the low eutectic point LiI–CsI mixture salts (217 °C) instead of the higher eutectic point LiCl–KCl mixture salts (353 °C) in the traditional LiM_x_–FeS_2_ thermal batteries.^[^ ^36^ ^]^ So the battery working temperature was reduced from 450–550 °C to 240–300°C. The sturdy and high‐density LLZTO tube can effectively separate molten lithium anode and fluid cathode at a high temperature. Meanwhile, tantalum‐doped LLZTO can provide higher Li^+^ conductivity than LLZO, as high as ≈0.085 S cm^−1^ at 250 °C and 0.13 S cm^−1^ at 300 °C.^[^ ^32^ ^]^

​The U‐shaped solid electrolyte tubes were first applied in high‐temperature liquid sodium metal batteries, such as sodium‐sulfur batteries (300–350 °C) and ZEBRA batteries (270–350 °C).^[^ ^37^, ^38^ ^]^ Both use metallic sodium anode and Na‐*β*‐Al_2_O_3_ or Na‐*β*"‐Al_2_O_3_ solid electrolytes. Reducing operating temperature and cost and increasing energy density have been the focus of liquid metal batteries.^[^ ^39^, ^40^ ^]^ Thus, Cui et al. developed solid electrolyte‐based liquid lithium (SELL) batteries (240–300 °C),^[^ ^32^, ^34^, ^35^, ^41^, ^42^ ^]^ which use metal lithium anode and U‐shaped solid electrolyte LLZTO. A U‐shaped battery structure ensures uniform transmission of Li⁺ and thus prevents excessive local current. Electrochemical numerical simulations for solid electrolyte‐based Li–FeS_2_ secondary batteries were performed to observe the Li^+^ transport and distribution during battery discharging and charging.^[^ ^43^ ^]^ Figure 1c shows the distribution of electrochemical potential in electrolytes and the current density vector (red arrows) when the battery voltage equals ≈2.1 V. Lithium ions tend to flow from the high electrochemical potential phase to the low electrochemical potential phase. It was shown that Li⁺ could uniformly migrate between the anode and cathode across the direction perpendicular to the walls of the LLZTO tube. Uniform Li^+^ migration will increase the ability to withstand large currents. Past experimental evidence shows that the LLZTO tube can withstand a high current of 500 mA cm^−2^.^[^ ^32^ ^]^ Even so, the lithium transfer pressure appears to be larger at the bottom of the ceramic tube than at the sides. Further numerical simulation (Figure S3, Supporting Information) shows that a design with a circular inner bottom of a positive collector should be able to counteract this.

The newly assembled Li–FeS_2_ secondary thermal battery had a ≈2.25 V open‐circuit voltage. Figure 1d shows the voltage‐specific capacity curve of a solid electrolyte‐based FeS_2_ secondary thermal battery with 200 mg FeS_2_ during the first two cycles. The discharge specific capacity of the first cycle was 800 mAh g^−1^. Subsequently, the battery had a little capacity loss, and the charge specific capacity of the first cycle was 764 mAh g^−1^. In the second cycle, the battery discharge curve was more stable, and the capacity was still as high as 756 mAh g^−1^, which is no sharp drop like a primary Li–FeS_2_ battery. The cyclic voltammetry (CV) curve proves good reaction reversibility between 1.2 and 2.3 V (Figure 1e). Two reduction peaks appeared during battery discharging, recorded as reduction peak I (≈1.95 V) and reduction peak II (≈1.6 V). Two oxidation peaks also appeared when battery charging: oxidation peak I (≈1.75 V) and oxidation peak II (≈2.2 V). These four peaks correspond to the redox of element Fe and the redox of element S, respectively.

### 2.2 From Fe and Li_2_S to FeS_2_: The Origin of High Reversibility

**Figure 2:**
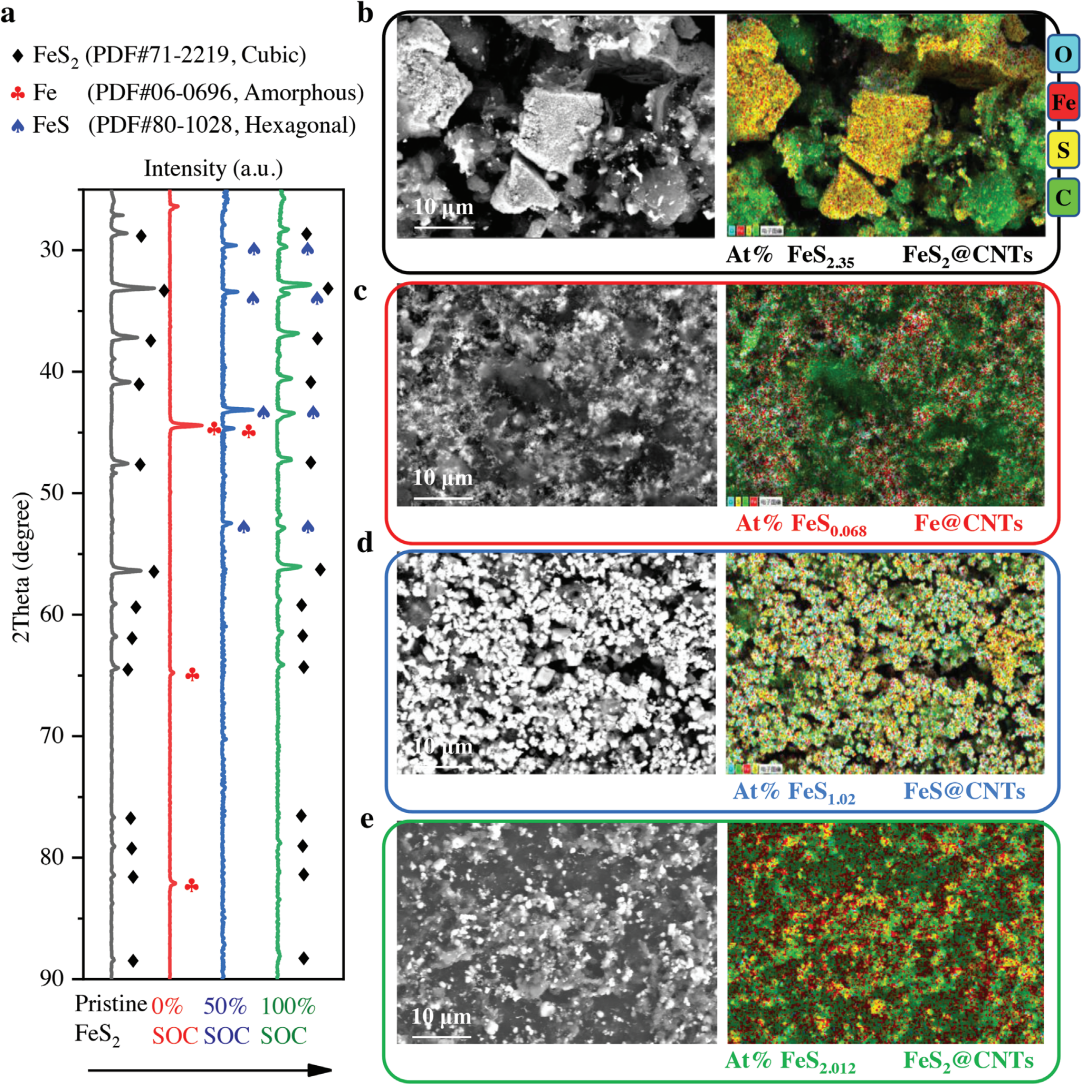
Characterization of battery materials and verification of reaction mechanism. a) Comparison of XRD curves before cycling and at 0%, 50%, and 100% SOC in the first cycle. b–e) SEM and EDS images of the battery cathode in different processes.

XRD tests for the battery cathodes of four states at the first cycle revealed the composition change during the battery reaction (**Figure** **2**a). The samples were washed with ultra‐pure water to remove water‐soluble LiI–CsI salts before the XRD testing. In this process, water‐soluble Li_2_S is also removed, making Li_2_S undetectable. FeS, FeS_2_, S, and Fe are insoluble in water and, therefore, can be detected if present^[^ ^44^, ^45^ ^]^ (Experimental Section). The red XRD pattern shows that the final reduction product contains elemental Fe because Fe (Ref. PDF # 06–0696) appeared in 0% SOC cathode. The FeS and Fe were detected in 50% SOC sample, which means that part of Fe has been oxidized into FeS (Ref. PDF # 80–1028). When the battery was recharged to 2.3 V with 100% SOC, FeS_2_, and FeS were detected, while the S‐peak did not appear. The peaks of FeS_2_ (Ref. PDF # 71–2219) after one cycle are the same as FeS_2_ before cycling. This implies that the final oxidation product at high temperatures after the cycle may be FeS_2_ but not S. The reaction process in the first cycle may be as follows: FeS_2_ is first lithiated to form Fe and Li_2_S (0% SOC), then Fe and Li_2_S delithiated to FeS and Li_2_S (50% SOC), and then delithiated to FeS_2_ (100% SOC).

Figure 2b–e shows the SEM and EDS images of the FeS_2_ cathodes under different battery states to compare the change in morphology and composition. The distributions of O, Fe, S, and C elements were observed in EDS tests, and the atomic ratios (At%) of S and Fe were used to figure out the main ingredients in the cathodes. The particle size of the FeS_2_ power initially is ≈40 µm. After grinding it with CNTs for 5 min, the size of FeS_2_ particle was reduced, and FeS_2_ surface was covered with lots of CNTs (Figure S4, Supporting Information). Then we melt FeS_2_@CNTs with lithium salts at 300 °C for 2 h. After cooling, the mixed block was crushed and used as the cathode. ​The samples used for the SEM and EDS tests were identical to those used for the XRD test, and all were washed with ultra‐pure water, thus removing lithium salts and Li_2_S. Before cycling, the At% of S: Fe is ≈2.35. When discharged to 1.2 V, the S–Fe atomic ratio is ≈0.068, and Li_2_S is basically washed away by ultra‐pure water, leaving only Fe in the sample. When charged to 1.8 V, the at.% of S: Fe is ≈1.02, of which the main is FeS. When continually charged to 2.3 V, the at.% of S: Fe is ≈2.012. Combined with the XRD patterns at 100% SOC, it can be seen that FeS_2_ is dominant at this time. After ≈500 h of cycling and discharge to 1.8 V, the at.% of S: Fe is ≈1.18 (Figure S5, Supporting Information).

No intermediate Li_2_FeS_2_ was observed in the above tests, but FeS is present in the cooled intermediate at 50% SOC, as evidenced by XRD, EDS, and SEM measurements. Neither Li_2_FeS_2_ nor FeS affects our overall understanding of these reactions since metastable Li_2_FeS_2_ has the same element ratio as FeS and Li_2_S (1:1). The ex situ technique we used is limited in its ability to capture metastable intermediates, as metastable Li_2_FeS_2_ may easily decompose into FeS and Li_2_S during sample preparation and measurement. We look forward to further studies of metastable intermediates at high temperatures using fully in situ characterization techniques. The four reactions of the battery, using FeS and Li_2_S as intermediates and corresponding to the four peaks of the CV curve, are shown as follows:

(1) $$\mathrm{Reduction}/\mathrm{Lithiation}\;\mathrm{reaction}\;I:{\mathrm{FeS}}_{2}+2\mathrm{Li}\;\overset{\mathrm{Discharge}}{\to }\mathrm{FeS}+{\mathrm{Li}}_{2}S\left(E\approx 1.95V\right)$$

(2) $$\mathrm{Reduction}/\mathrm{Lithiation}\;\mathrm{reaction}\;\mathrm{II}:\mathrm{FeS}+2\mathrm{Li}\;\overset{\mathrm{Discharge}}{\to }{\mathrm{Li}}_{2}S+\mathrm{Fe}\left(E\approx 1.6V\right)$$

(3) $$\mathrm{Oxidation}/\mathrm{Delithiation}\;\mathrm{reaction}\;I:{\mathrm{Li}}_{2}S+\mathrm{Fe}\;\overset{\mathrm{Charge}}{\to }\mathrm{FeS}+2\mathrm{Li}\left(E\approx 1.75V\right)$$

(4) $$\mathrm{Oxidation}/\mathrm{Delithiation}\;\mathrm{reaction}\;\mathrm{II}:{\mathrm{Li}}_{2}S+\mathrm{FeS}\;\overset{\mathrm{Charge}}{\to }{\mathrm{FeS}}_{2}+2\mathrm{Li}\left(E\approx 2.2V\right)$$

**Figure 3:**
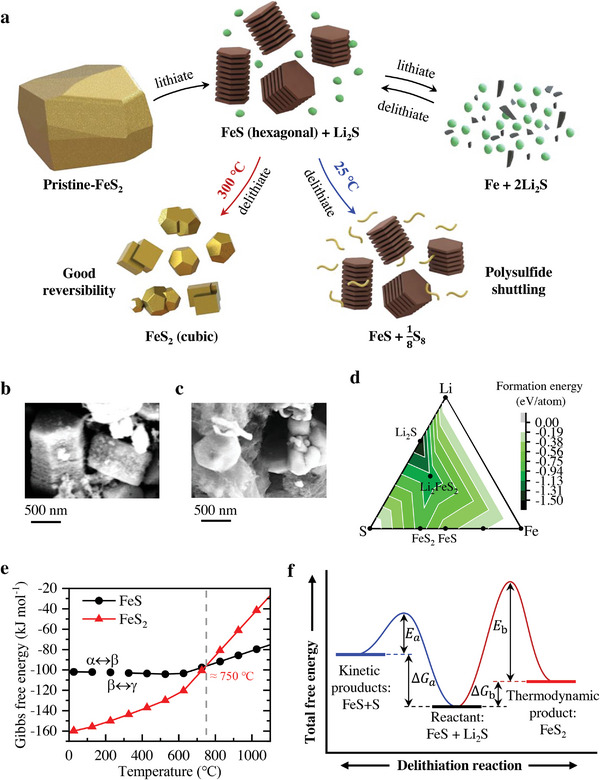
The reaction mechanism of high‐temperature Li‐FeS_2_ batteries. a) Schematic of lithiate and delithiate process of the cathodes at 25 and 300 °C. b) Hexagonal and layered FeS in 1.8 V cathode. c) Cubic FeS_2_ particles in 2.3 V cathode. d) Li–S–Fe ternary phase diagram and formation energy distribution. e) Gibbs free energies of FeS and FeS_2_ under different temperatures. f) The change of oxidation products due to temperature‐induced kinetic and thermodynamic competition.

Associated with the above equations, we draw the schematic of the lithiate and delithiate process of the cathode at 25 and 300 °C in **Figure** **3**a. It can be seen that the reaction mechanism of the Li–FeS_2_ battery at 300 °C is different from 25 or 60 °C.^[^ ^5^ ^]^ The final oxidation product at 25 or 60 °C is S and FeS, whereas at 300 °C is FeS_2_. The difference in products would directly affect the reversibility of the Li–FeS_2_ batteries because the formation of sulfur would bring about the notorious shuttle problem, which is also a major difficulty for Li–S batteries. The morphology of the intermediate FeS is consistent with the XRD results, and it is a layered structure of a hexagonal crystal system (Figure 3b). Furthermore, FeS_2_ is a cubic crystal, mainly showing a regular dodecahedron, a cube, and their stacking structure (Figure 3c). The predicted formation energies were given in the ternary phase diagram of Li–S–Fe at 0 K in Figure 3d.^[^ ^46^ ^]^ The formation energies of elemental substances are ≈0, and the formation energy of Li_2_S is the smallest, which is –1.504 eV atom^−1^. This is consistent with the state change during the reactions. The lithium intercalation process from FeS_2_ to Li_2_S is discharged and spontaneous, while the transition from Li_2_S to FeS_2_ requires additional energy provided by charging.

Working temperature (*T*) plays a key role in the choice of the final oxidation product. As shown in Figure 3e, the Gibbs free energy (*G*) of FeS rises very slowly due to two solid‐state phase transitions of FeS, both associated with electronic magnetic phenomena.^[^ ^47^ ^]^ The adopted temperature of the *α* → *β* transition is 137.85 ± 3 °C, and the *β* → *γ* transition is 324.85 ± 3 °C. When the temperature is <750 °C, the reaction of FeS + S → FeS_2_ is spontaneous in thermodynamics. The reactant FeS and Li_2_S formed FeS and S after delithiating at room temperature, whereas FeS_2_ formed after delithiating at high temperature. The latter may have higher activation energy (*E*_b_ *> E*_a_) and thus be kinetically disadvantaged. According to the Arrhenius equation: $k\;=\;Ae^{-E_{a}/RT}$, the effect of activity energy *E*_a_ on the rate constant *k* can be reduced by increasing the temperature *T. R* is the universal gas constant. As a rough estimate, the reaction rate increases about two or three times for every 10 °C increase. It is therefore reasonable to speculate that FeS and S are kinetic products that are more easily produced at low temperatures. At the same time, FeS_2_ is a thermodynamic product that is easier to produce at high temperatures. The high‐temperature reactions are from FeS_2_ to Fe and Li_2_S at discharge and back to FeS_2_ after charging. There exists a temperature range for FeS_2_ regeneration, which is roughly estimated at 197–750 °C, where 197 °C is the reported temperature for FeS + S → FeS_2_^[^ ^27^ ^]^ and 750 °C is the decomposition temperature of FeS_2_ thermodynamically. Currently, the operating temperature range of SELL batteries is 240–300 °C, which is in the range of 197–750 °C. Thus these operating temperatures can guarantee the reversible reactions in Li‐FeS_2_ batteries from Fe and Li_2_S to FeS_2_.

Considering the influence of reaction kinetics, two oxidation reactions or two discharge reactions may proceed simultaneously. As shown in Figure S6 (Supporting Information), the introduction of the coefficients *x* and *y* is to describe the reaction extent more accurately in the actual situation. Assuming that the initial FeS_2_ quantity is 1, *x* + *y* ≤1, *x* ≥ 0, and *y* ≥ 0. The *x* and *y* would change with the reaction conditions, such as the battery rate and battery working temperature. However, in a real battery working condition, the *x* and *y* values can be calculated by the actual specific capacity. The Li–FeS_2_ secondary thermal battery with 200 mg FeS_2_ in Figure 1d has *x* ≈ 0.67 and *y* ≈ 0.17, indicating that in the reduction reaction II, the main reactant is still FeS, but there have a small amount of FeS_2_ involved, which is different from that of room temperature Li–FeS_2_ battery ($\mathrm{FeS}+2\mathrm{Li}\overset{\mathrm{Discharge}}{\to }{\mathrm{Li}}_{2}S+\mathrm{Fe}$). At the same time, the FeS_2_ utilization is equal to (*x* + *y*) × 100%, equivalent to the ratio of the maximum specific capacity to the theoretical specific capacity (894 mAh g^−1^). Here, the FeS_2_ utilization is ≈84%, which is already high.

### 2.3 FeS_2_ Pulverization Phenomenon During Cycling

**Figure 4:**
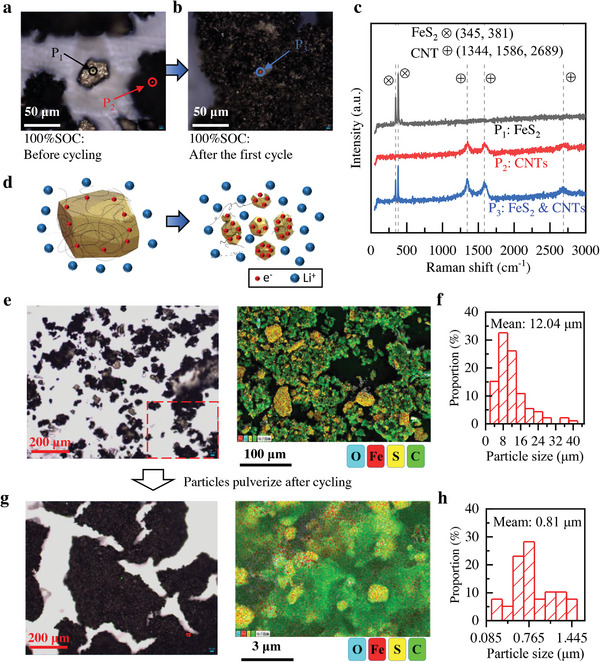
Particle pulverization in FeS_2_ cathodes. a,b) The test points in Raman tests of FeS_2_ cathodes. c) Raman spectrums of P_1_, P_2_, and P_3_. d) More efficient contact after powdering in the liquid electrode. e) The large‐scale optical and EDS image of the cathode before cycling at 100% SOC. f) The mean particle size of FeS_2_ before cycling. g) The large‐scale optical and EDS image after cycling at 100% SOC. h) The mean particle size of FeS_2_ after cycling.

The optical images of the battery cathodes before and after cycling at 100% SOC show many yellow particles and black cotton‐like substances (**Figure** **4**a,b). Moreover, the size of the yellow particles before the cycle is significantly larger than after the cycle. Three points, P_1_ and P_2_ in Figure 4a and P_3_ in Figure 4b, were selected for the Raman test. Raman spectroscopy results showed that P_1_, P_2_, and P_3_ are FeS_2_, CNT, and FeS_2_ and CNT, respectively (Figure 4c). After one battery cycle, both FeS_2_ and CNTs were detected at a small point P_3_. This means that the pristine FeS_2_ particles were severely pulverized after cycling and were in better contact with the CNTs. In addition, Raman spectroscopy verified that the final oxidation product is FeS_2_, in agreement with the results of the XRD and EDS tests described above.

Conversion reactions in organic electrolyte‐based Li–FeS_2_ batteries lead to severe volume expansion and electrode pulverization issues, but solid electrolyte‐based Li–FeS_2_ batteries can mitigate these adverse effects. The FeS_2_ particle size before cycling is about tens of microns, which would be significantly reduced after cycling and become nano‐scale Fe, FeS, or FeS_2_ in different reaction stages (Figures S7–S9, Supporting Information). As shown in Figure 4d, the particle pulverization of the positive active material favors the transport of Li⁺ and e^−^ at the cathode, as the flowing positive electrode slurry instantly coats the pulverized particles, which improves the contact effect between the positive active material and the CNTs and lithium salts. Simultaneously, the sturdy LLZTO tube makes the battery structure stable when the active material expands.

Figure 4e,g shows large‐scale optical photographs of the cathodes before and after the cycle. After cycling, the smaller FeS_2_ had a better agglomeration with the CNTs. The distribution of FeS_2_ particles and CNTs can be compared by performing an EDS test on the cathode before and after the cycling, detecting Fe, S, O, and C elements. If ignoring the scale ruler, the distribution situations of FeS_2_ particles (red and yellow) surrounded by CNTs (green) before and after cycling are almost the same. But the scales are different, which are 100 µm before cycling and 3 µm after cycling. In Figure 4e,g, the red frames are used to mark the area size of the EDS test to more intuitively compare the FeS_2_ particle sizes, and are not the true positions in the EDS test. The distribution diagram of particle size in Figure 4f,h shows the particle size distribution of FeS_2_ particles, which decreases from ≈12.04 µm before cycling to ≈0.81 µm after cycling.

After a rough calculation, the diameter, surface area, and volume of the FeS_2_ particles before cycling are ≈15, 225, and 3375 times, respectively, than those after cycling. The severe pulverization problem of FeS_2_ materials rapidly reduces the capacity of room‐temperature Li‐FeS_2_ batteries after cycling, posing a huge obstacle to the development of Li‐FeS_2_ secondary batteries. ​Fortunately, the itinerant CNTs and lithium salts in the fluid cathode can encapsulate the pulverized FeS_2_ in time and increase the active region. In addition, molten salt electrolytes have fewer side reactions with active materials than organic electrolytes at room temperature. With fewer side reactions, the capacity does not decrease even though the specific surface area of the active material increases as the electrode is pulverized.

### 2.4 Electrochemical Performances of Li–FeS_2_ Secondary Thermal Batteries

**Figure 5:**
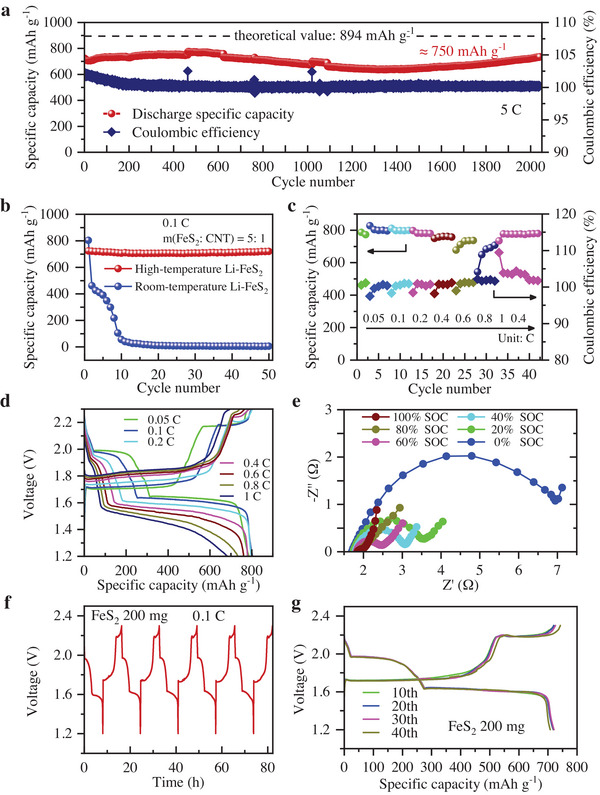
Electrochemical performances of Li–FeS_2_ secondary thermal batteries. a) 2000 cycles performance at 5 C. b) Performance comparison with organic electrolyte‐based Li–FeS_2_ battery. c) Rate performance. d) The specific capacity–voltage curves under different current rates. e) EISs under different SOCs. f) Voltage curve at 1–5 cycles. g) The specific capacity–voltage curves at the 10th, 20th, and 30th cycles.

Due to a series of advantages brought by the battery structure and the reversible reaction mechanism, solid electrolyte‐based Li–eS_2_ secondary thermal cells achieve highly reversible cycles and perform long time periods. As shown in **Figure** **5**a, it can reversibly cycle more than 2000 cycles at a rate of 5 C (3 mg FeS_2_, 13.4 mA) with the discharge specific capacity of ≈750 mAh g^−1^. Even as the running time increases, the battery performance improves. The failure of this battery occurred after 800 h at 300 °C. The reason is insufficient manufacturing processes resulting in weak areas in the thin (≈1 mm) and not very dense LLZTO tubes, where lithium corrodes the ceramic tubes at high temperatures and deposits in the internal holes of the ceramic tubes.^[^ ^32^, ^35^ ^]^ Next, we can investigate high‐quality LLZTO fabrication and corrosion‐resistant coatings to significantly extend the life of SELL batteries. In contrast, the discharge specific capacity of the organic electrolyte‐based Li‐FeS_2_ battery (0.48 mg FeS_2_, 0.1 C, ≈43 µA, 25 °C) decreased rapidly as the cycle number increased and could not discharge after just ten cycles (Figure 5b). Meanwhile, the voltage curve of the organic electrolyte‐based Li–FeS_2_ battery is not as stable as the solid electrolyte‐based Li‐FeS_2_ battery (Figure S10, Supporting Information).

After verifying the reversible cycle performance, a larger capacity Li–FeS_2_ secondary thermal battery with 20 mg FeS_2_ was fabricated to test the rate performance at different charge–discharge rates. The rates gradually increased from 0.05 C (0.9 mA) to 1 C (17.8 mA) and then reduced to 0.4 C (7.2 mA). The rate performance result showed that the battery could maintain a stable Coulombic efficiency and exert a large specific capacity under different rates (Figure 5c). When the charge–discharge rate is changed, the Coulombic efficiency can be restored to ≈100% after a fast adjustment.

In Figure 5d, the discharge specific capacity–voltage curve under different current rates showed that the voltage platform, discharge medium voltage, and proportion of each reaction stage (expressed by *x*, *y*) would change with the current rate change. In general, as the current rate increases, the charging platform increases and the discharging platform decreases. Discharge medium voltage, a voltage when the battery discharge capacity is half of the maximum discharge capacity, decreases with the increase of the current rate (Figure S11, Supporting Information). Interestingly, the discharge medium voltage at 0.4 C after 32 cycles is higher than that at 0.4 C during 13–17 cycles, which means that the battery performance improves with the number of cycles. For the change of coefficients *x* and *y* of battery reactions under different current rates (Figure S6, Supporting Information), *x* decreases, and *y* increases with the current rate increase (Figure S12, Supporting Information). As the current rate increases, FeS_2_ tends to form Fe directly while omitting FeS intermediate states.

Moreover, some large‐capacity Li–FeS_2_ secondary thermal batteries with 200 mg FeS_2_ and 180 mg Li were fabricated to test the EISs and lithium utilization. The EISs under different SOCs are different (Figure 5e). The resistance decreases with increasing SOC, which is due to the gradual reduction of the poorly conducting Li_2_S with increasing SOC. The battery resistance is ≈2 Ω at 100% SOC. The battery can effectively reversibly cycle at 0.1 C (17.8 mA) with a reversible capacity of ≈140 mAh. Considering that the theoretical specific capacity of lithium metal is 3860 mAh g^−1^, the lithium utilization in anode is ≈20%. The large‐capacity Li‐FeS_2_ battery has two discharging platforms (≈1.95 and 1.6 V) and two charging platforms (≈1.8 and 2.2 V) (Figure 5f). In addition, as the cycle number increases, the specific capacity–voltage curve of the battery is almost unchanged, indicating that the battery has a better cycle performance (Figure 5g).

## 3 Conclusion

In summary, we demonstrated a highly reversible solid electrolyte‐based Li‐FeS_2_ secondary thermal battery and clarified the reversible mechanism of Li‐FeS_2_ secondary thermal battery. The problem of pulverization of FeS_2_ particles was successfully solved due to the liquid‐solid structure of the FeS_2_ slurry cathode‐LLZTO tube anode. Moreover, the reaction mechanism of Li‐FeS_2_ at high temperatures is significantly different from that at room temperature. It achieves a reversible transition from Fe and Li_2_S to FeS_2_ at high temperatures. These successfully converted Li–FeS_2_ thermal batteries from primary to secondary. The assembled batteries can effectively cycle 2000 times at a rate of 5 C (≈4.5 A g^−1^). Meanwhile, it has a high reversible specific capacity of ≈750 mAh g^−1^ and a stable second discharge platform of ≈1.6 V. Benefiting from the low cost and good performance of FeS_2_ materials, the Li‐FeS_2_ secondary thermal batteries are expected to play an essential role in large‐scale energy storage and other harsh environments such as deserts and outer space.

## Conflict of Interest

The authors declare no conflict of interest.

## Supporting information

Supporting Information

## Data Availability

The data that support the findings of this study are available in the supplementary material of this article.
