# Supplementary material for: Modulation of the Oxidation End‐Product Toward Polysulfides‐Free and Sustainable Lithium‐Pyrite Thermal Batteries
Source: Adv Sci (Weinh). 2023 Jan 5;10(6):2205888. doi: 10.1002/advs.202205888 (PMC9951353; doi:10.1002/advs.202205888)
Supplement: Supplementary file 1 — Supporting Information [file ADVS-10-2205888-s001.pdf]

Supplementary Information for

Modulation of the Oxidation End-product toward Polysulfides-free and Sustainable Lithium-pyrite Thermal Batteries

Yang Jin[1*], Hongfei Lu[1], Nawei Lyu[1], Di Zhang[1], Xin Jiang[1], Bin Sun[1], Kai Liu[2*], Hui Wu[2*]

Prof. Y. Jin, Dr. H. Lu, Dr. N. Lyu, Mr. D. Zhang, Dr. X. Jiang, Dr. B. Sun
Research Center of Grid Energy Storage and Battery Application, School of Electrical Engineering, Zhengzhou University, Zhengzhou, Henan 450001, China
E-mail: yangjin@zzu.edu.cn, luhfff@126.com, naweilyu@zzu.edu.cn, zhangdi3512@163.com, jiangxin@zzu.edu.cn, binsun@zzu.edu.cn
Prof. K. Liu
State Key Laboratory of Alternate Electrical Power System with Renewable Energy Sources, School of New Energy, North China Electric Power University, Beijing 102206, China.
E-mail: l-k08@163.com
Prof. H. Wu
State Key Lab of New Ceramics and Fine Processing, School of Materials Science and Engineering, Tsinghua University, Beijing 100084, China
E-mail: huiwu@tsinghua.edu.cn

Experimental

Preparation of the LLZTO tubes. Li_2CO_3 (Sinopharm Chemical Reagent Co., Ltd, 99.99%), La_2O_3 (Sinopharm Chemical Reagent Co., Ltd, 99.99%), ZrO_2 (Aladdin, 99.99%), and Ta_2O_5 (Ourchem, 99.99%) powders were mixed at a molar ratio of $Li_{6.5}La_3Zr_{0.5}Ta_{1.5}O_{12}$, ground with an agate mortar and pestle, and then heated at 900 °C for 6 hours to decompose the metal salts. The resulting powders were ball milled for 12 hours before being pressed into a tube with cold isostatic pressing at 330 MPa for 120 s and then annealed at 1140 ^{o}C for 16 hours in the air. At the same time, the tube was covered with the same mother powder to replenish lithium when heating. All heat treatments were conducted in alumina crucibles (> 99% Al_2O_3) covered with alumina lids. The Archimedes ethanol displacement method was used to measure the relative density of the LLZTO garnet tube. The average density of the LLZTO tube can reach 5.2 g cm^{-3} (relative density reaches 96.3 %).

Preparation of LiI-CsI lithium salts. Weigh the LiI (Shanghai China Lithium Industrial Co., Ltd, > 99.9%) and CsI (Shanghai China Lithium Industrial Co., Ltd, > 99.9%) salts according to the molar ratio of LiI: CsI of 0.662 to 0.338, which is the mass ratio of LiI: CsI is 8.86: 8.78. Pour the two into the quartz crucible and stir evenly. Put a lid on the quartz crucible and place them in the box furnace. The temperature is raised to 350 °C in 1 hour and kept at 350 °C for 2 hours. After cooling to room temperature, take out the salt block and crush it. All operations above are carried out in a glove box (H_2O and O_2 < 0.01 ppm) to prevent the salt from deliquescing.

Preparation of FeS_2 cathode. The cathode of the solid electrolyte-based Li-FeS_2 secondary thermal battery comprises FeS_2 (Alfa Aesar, 99.99%), CNT (XFNANO Co., Ltd, diameter: 5-15 nm, length: 10-30 μm), and LiI-CsI lithium salts. First, weigh FeS2 powder and CNT, then grind them together for 5 minutes. Pour the CNT, and FeS_2 mixed powder into a quartz crucible, then pour the prepared LiI-CsI mixed salts into the quartz crucible, then put the quartz crucible in the box furnace in the glove box (H_2O < 0.01ppm, O_2 < 0.01ppm), heat them to 300 °C in 40 mins and keep at 300 °C for 2 hours. After naturally cooling, the three would form a solid mixture used for the cathode, in which FeS_2 and CNT are uniformly dispersed in the lithium salts. The ratio between the FeS_2, CNT, and LiI-CsI lithium salts greatly affects the performance of the solid electrolyte-based Li-FeS_2 secondary thermal battery. Our self-made battery case can contain up to ≈ 3 g of lithium salts, and filling it full with lithium salts can maximize the contact area of the liquid-solid interface. There are three battery cathode ratios in

this paper. The Li-FeS_2 battery used for 5 C rate contains 3 mg FeS_2, 60 mg CNTs, 3 g lithium salts, respectively. The larger Li-FeS_2 battery used for the rate test contains 20 mg FeS_2, 10 mg CNTs, and 600 mg lithium salts, respectively. Large-capacity Li-FeS_2 secondary thermal batteries contain 200 mg FeS_2, 40 mg CNT, and 2.4 g lithium salts in the cathode. The proportion of CNTs in the lithium salts affects the fluidity of the cathode slurry, thereby affecting the Li^+ transmission efficiency. It found that the smaller the mass ratio of CNTs to lithium salts, the better the conductivity of Li^+. The mass ratio of CNTs to FeS_2 affects the electron conduction of the cathode, thereby affecting the discharge specific capacity. The larger the mass ratio of CNTs to FeS_2, the better the e^- conductivity in the cathode and the higher the specific capacity.

Fabrication and characterization of solid electrolyte-based Li-FeS_2 secondary thermal batteries. Shatter the FeS_2 cathode block, put the powder into the 316 L stainless steel battery case, and install the Al_2O_3 insulator cover. Wind the lithium sheet (160~180 mg) into a long cylindrical shape, insert it into the LLZTO tube and then insert the negative collector into the lithium anode. Put the LLZTO tube from the hole of the Al_2O_3 insulator cover into the battery case. Put the battery in a box furnace and heat it to 300 °C. As the heating progresses, the solid cathode would melt, and the LLZTO tube gradually sinks and finally gets stuck by the hole of the Al_2O_3 insulator so that a solid electrolyte-based Li-FeS_2 battery is completed. The assembly and testing of the battery are carried out in a glove box (H_2O < 0.01 ppm, O_2 < 0.01 ppm). XRD (MiniFlex600-C with Rigaku) was used to monitor the phase formation of the cathode. RAMAN spectrometer (Xplora Plus with Horiba) was used to perform Raman spectroscopy. SEM (Auriga-bu with Zeiss) is used to analyze the electrode structure, and its subsidiary function EDS is used for elemental analysis. Battery test system (LAND 2001 CT battery tester) to test the electrochemical performances of the solid electrolyte-based Li-FeS_2 battery at 300 °C. An impedance analyzer (VSP with Bio-Logic) was used to measure the electrochemical impedance spectroscopy.

Remove the lithium salts in the samples before XRD and SEM tests. The method to remove LiI-CsI lithium salts is as follows: ground the cathode into fine powders, pour the powders into ultrapure water, ultrasound for ≈ 5 mins, use a 0.45 μm water-based filter paper for vacuum filtration, dry the filter paper in the oven, scrape off the powder. It should be noted that Li_2S would dissolve in water and be washed away, and the elemental S would not dissolve in water.

Assembly and test of organic electrolyte-based Li-FeS_2 coin cell. To compare

the performance with solid electrolyte-based FeS_2 batteries, we have produced organic electrolyte-based FeS_2 batteries operating at 25 °C. The size of FeS_2 particles would affect the battery performance greatly, so we use electrochemical methods to prepare nano-FeS_2 particles. The method of preparing nano-FeS_2 particles is to completely pulverize the FeS_2 particles in the solid electrolyte-based Li-FeS_2 battery after several cycles and then use ultrapure water to completely remove the lithium salts in the cathode to obtain a mixture of nano-FeS_2 and CNT with a mass ratio of 5: 1. First, The PVDF is uniformly dispersed in NMP by magnetic stirring. The mass of PVDF is a quarter of the total mass of nano-FeS_2 and CNT, and about 10 μL of NMP is required for every 1 mg of PVDF. Then, add nano-FeS_2 and CNT powder to the PVDF-containing NMP solution, and stir them for 8 h. Then paint the slurry on aluminum foil (carbon-coated Al foil, expressed as C@Al, purchased from Hefei Kejing Instrument Co., Ltd.), flat with a 25 μm scraper, and place it in a vacuum oven at 80 °C for 12 hours. Finally, it is cut into pellets with a diameter of 12 μm, and the FeS_2 cathode pellet has an active material load of ~ 0.4 mg cm^{-2}. The organic electrolyte-based FeS_2 coin cell is made by the lithium anode pellet, PP separator, FeS_2 cathode, and a large amount of electrolyte (~ 60 μL, 1 M $LiPF_6$ in EC: DEC, Aladdin). Then use a coin cell press to compress the 2032 battery case under a pressure of about 500 psi. The organic electrolyte-based FeS_2 coin cells stand for 8 hours to wet the electrodes fully before testing. The organic electrolyte Li-FeS_2 battery in Fig. 4b is a coin cell run at 0.1 C (~ 43 μA) at room temperature (25 °C), in which the masses of FeS_2, CNT, and PVDF in the cathode are 0.48 mg, 0.1 mg, and 0.15 mg, respectively.

Supplementary Figures

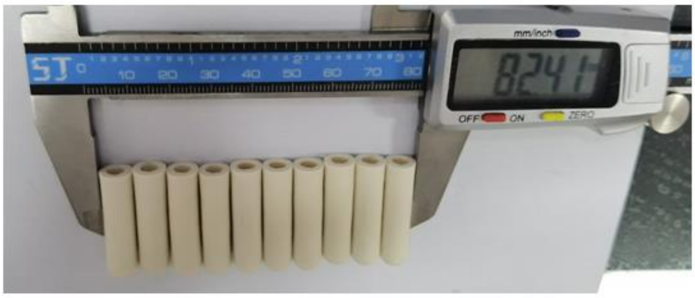

Figure S1. U-shaped LLZTO tubes

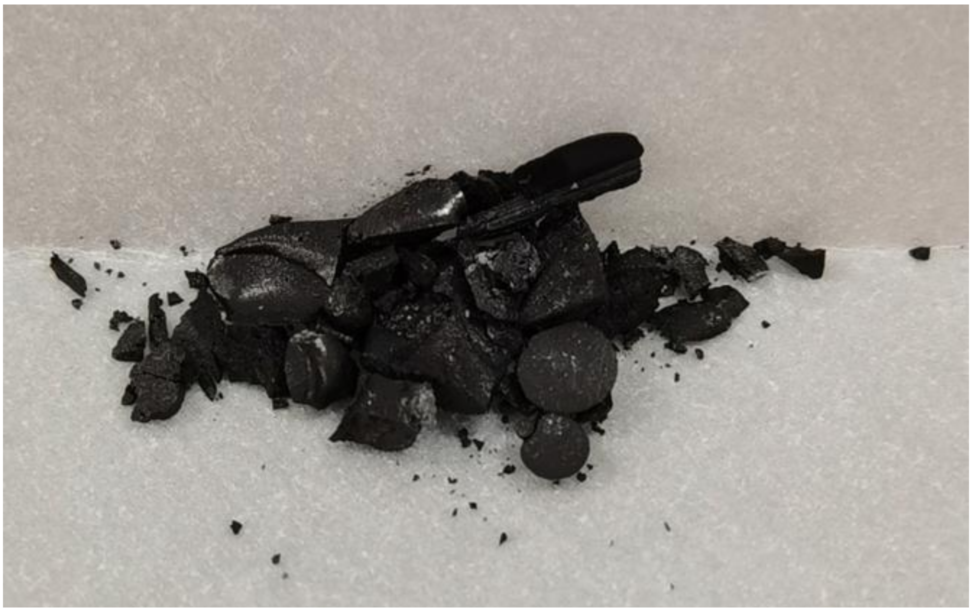

Figure S2. The photograph of the FeS_2 cathode used in the Li-FeS_2 secondary thermal battery, composing FeS_2, CNTs, and lithium salts.

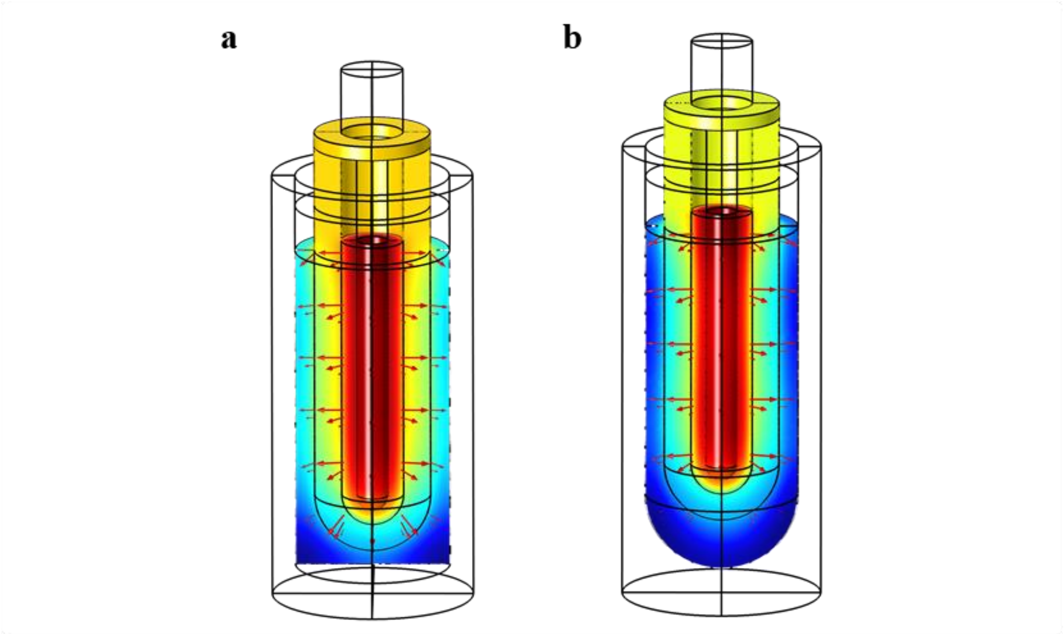

Figure S3. Numerical simulation results of electrochemical potential in electrolytes and current density vector during battery discharging at ≈ 2.1 V. a) Square inner bottom of the positive collector. b) Circular inner bottom of the positive collector.

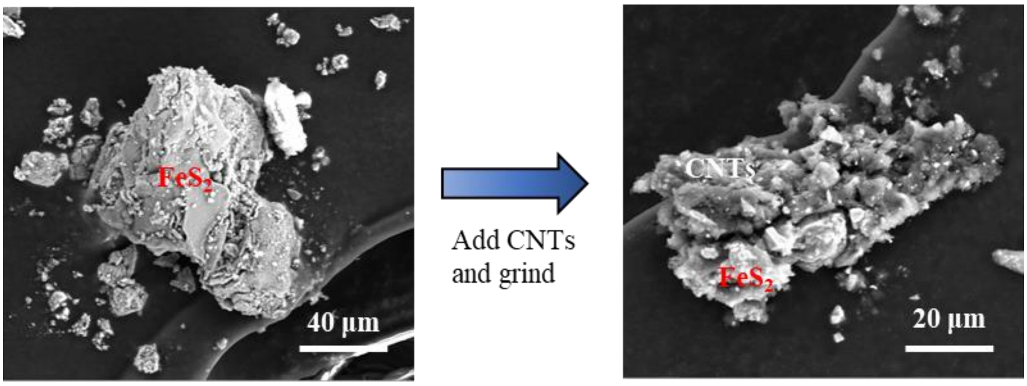

Figure S4. FeS_2 before and after grinding with CNTs.

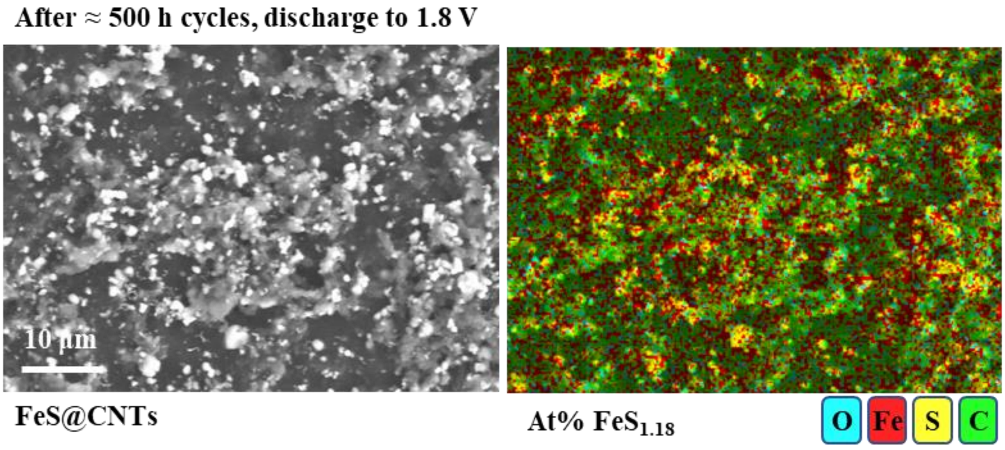

Figure S5. SEM and EDS image of the battery cathode at 1.8 V after ≈ 500 h cycles.

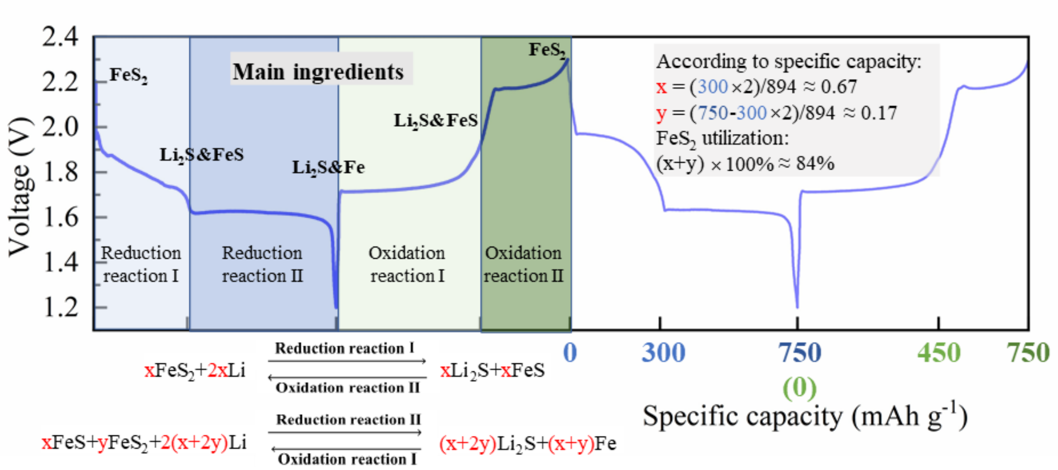

Figure S6. The main ingredients in each stage and the more accurate reaction formulas considering the effect of reaction kinetics.

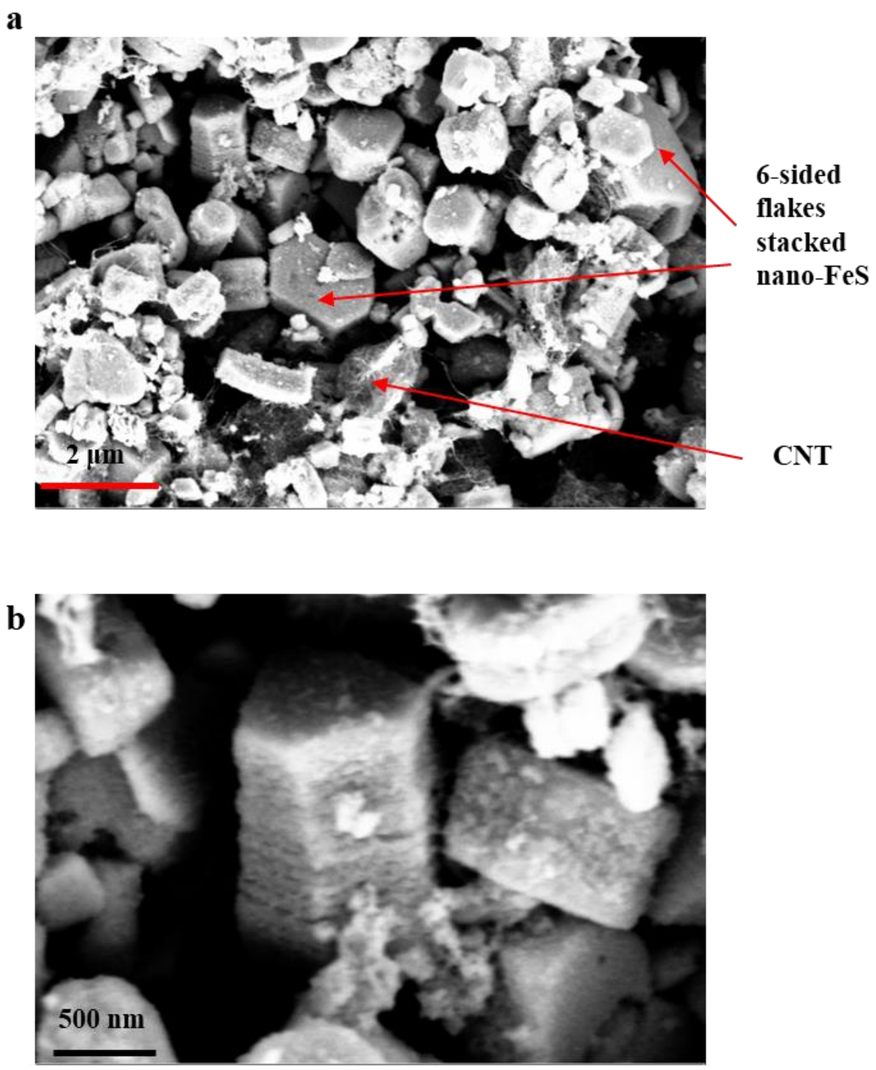

Figure S7. The micro-morphology of the cathode of the solid electrolyte-based Li-FeS_2 battery at 1.8 V, in which the lithium salts have been removed by ultra-pure water.

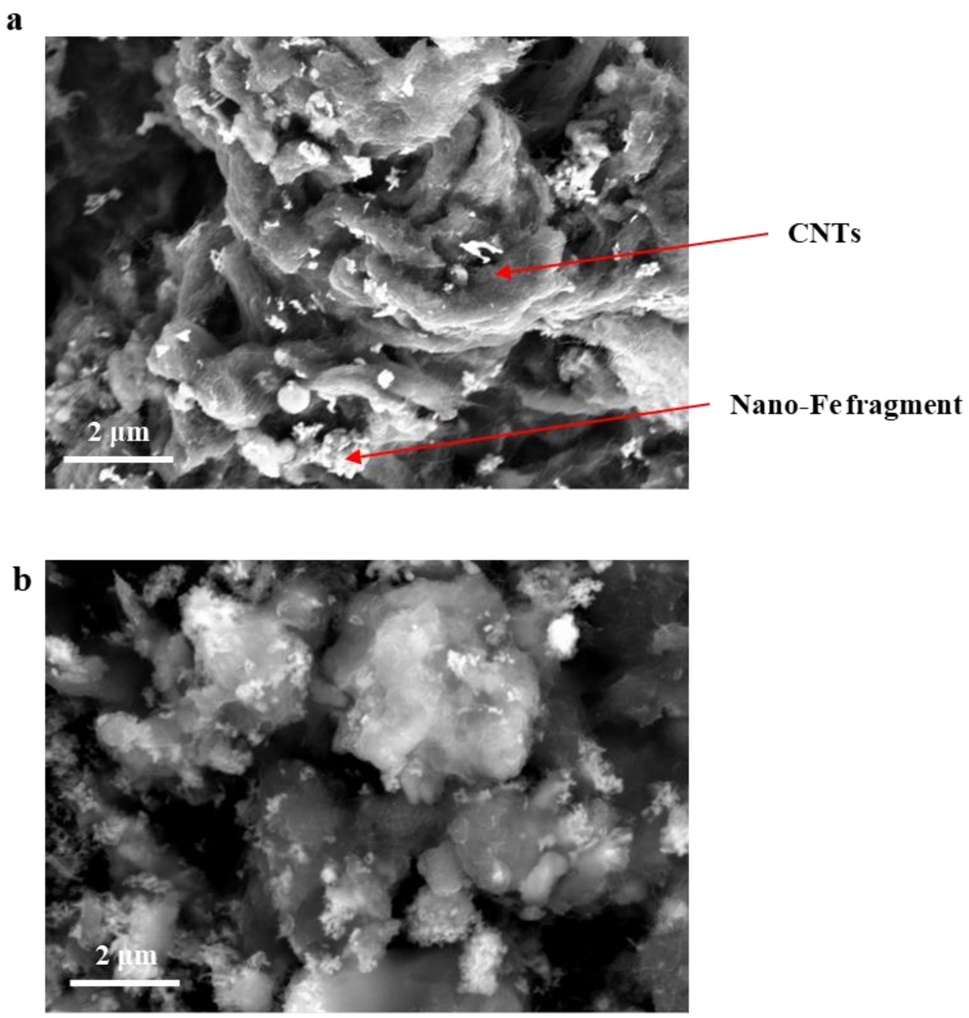

Figure S8. The micro-morphology of the cathode of the solid electrolyte-based Li-FeS_2 battery at 1.2 V, in which the lithium salts have been removed by ultra-pure water. Many irregular nano-Fe fragments are embedded in bulk CNTs to form μm-scale spherical clusters.

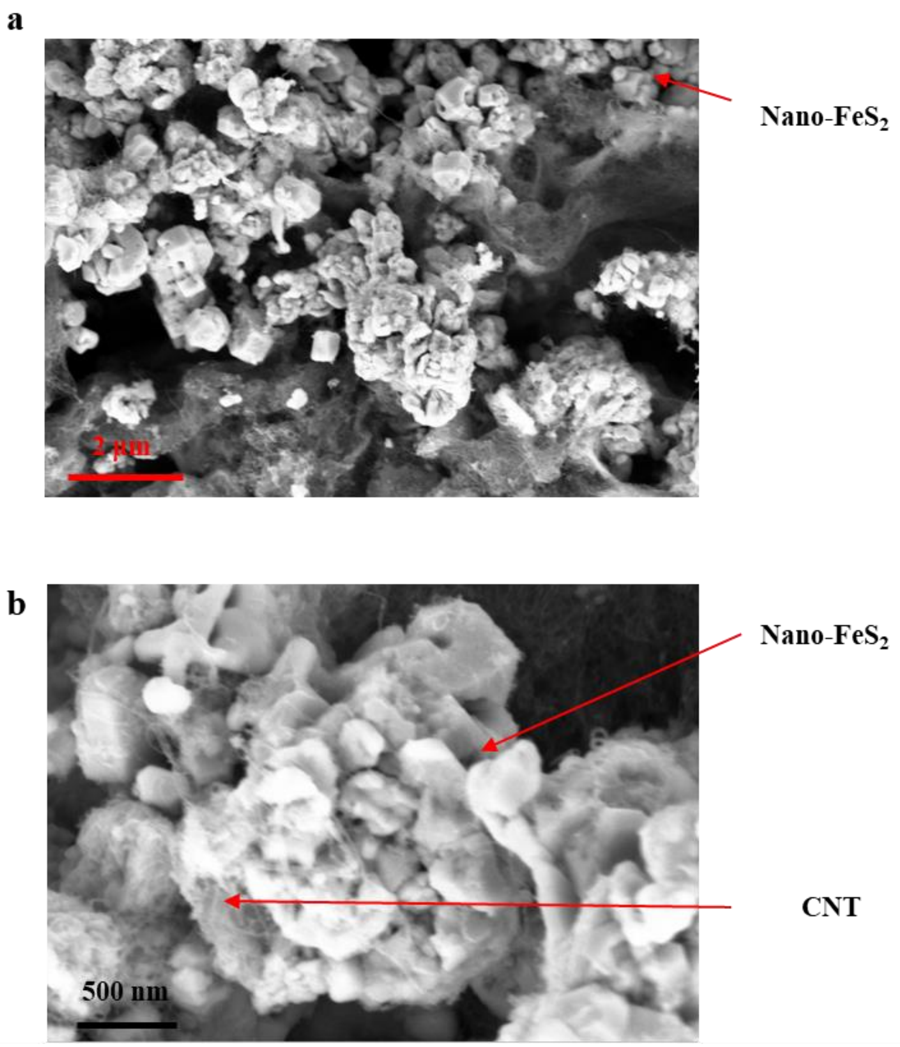

Figure S9. The micro-morphology of the cathode of the solid electrolyte-based FeS_2 battery at 2.3 V, in which the lithium salts have been removed by ultra-pure water. We see many nano-FeS_2 particles surrounded by CNTs.

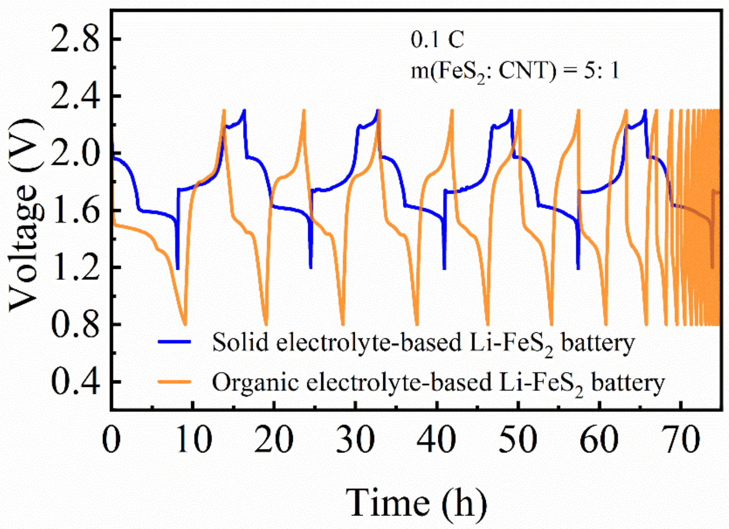

Figure S10. The comparison of voltage curves of an organic electrolyte-based Li-FeS_2 battery and a solid electrolyte-based Li-FeS_2 battery with the same current rate and mass ratio of FeS_2 and CNTs. The discharge platform of the organic electrolyte Li-FeS_2 battery is lower than that of the solid electrolyte-based Li-FeS_2 battery.

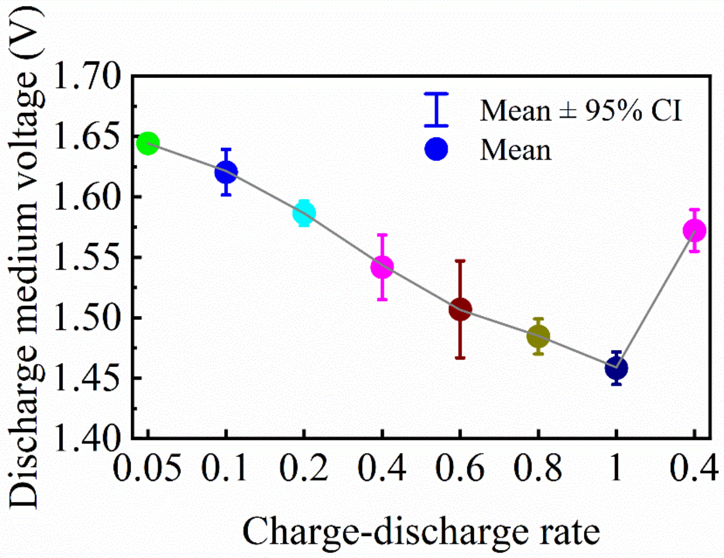

Figure S11. The change of discharge medium voltage of Li-FeS_2 secondary thermal battery with 20 mg FeS_2 under different charge-discharge rates.

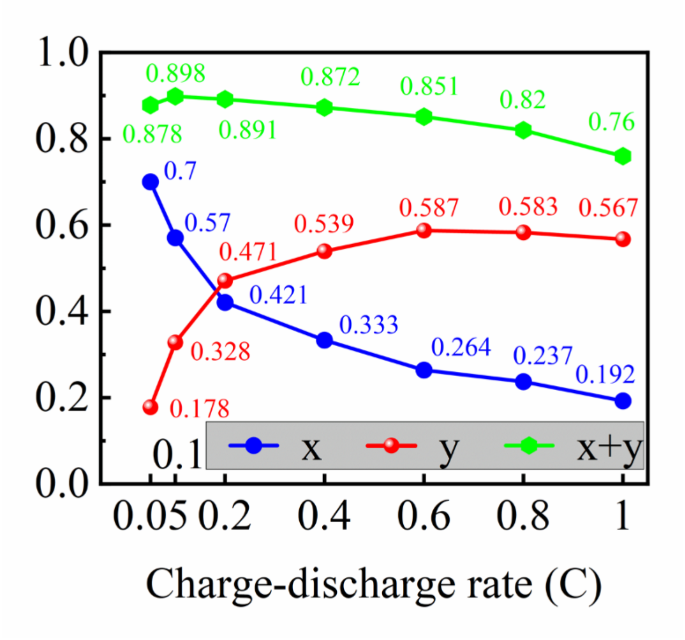

Figure S12. The values of x, y, and (x+y) under different charge-discharge rates.
